# H7N9 flu

**DOI:** 10.1186/1471-2334-14-S2-S21

**Published:** 2014-05-23

**Authors:** Bruno Lina

**Affiliations:** 1 National Research Center on Influenza Virus, Hospices Civils of Lyon, Claude Bernard University, Lyon, France

## 

Aquatic wild birds are the reservoir of more than 100 influenza viruses whose subtypes (HxNy) have never been responsible for sustained infection in humans. As a matter of fact, humans are usually not infected by avian influenza A viruses. However, sporadic cases of avian influenza virus infection are observed every year, mostly in Asia. By subsequent adaptation these emerging viruses may result into human pandemic strains.

Recently, a novel influenza A virus of the H7N9 subtype has been isolated from severely diseased patients with pneumonia and acute respiratory distress syndrome and, apparently, from healthy poultry in March 2013 in Eastern China. This virus is a result of a triple reassortment event that occurred in wild birds. Transmission to humans occurred in wet markets, mostly in large cities like Shanghai. Only a very limited number of cases have been observed out of China (Malasia, Tawain and Canada). As opposed to H5N1 that infect mostly children, most of the H7N9 cases were reported in adults and elderly people. As of the 7th of April, the death toll of this emergence is 30% (122/413). No human to human transmission has been reported so far. This lack of transmission cannot be explained by the virological features of the H7N9 strains that harbours most of the known markers required to facilitate transmission in mammals. In vitro investigations have shown that the recent A(H7N9) outbreak in Eastern China is due to a virus that replicates as efficiently as human-adapted influenza viruses. WHO experts consider this virus as an imminent threat.

